# Six Weeks of a Progressive Heavy Back-Squat Post-Activation Performance Enhancement Protocol and Countermovement Jump Performance in Competitive Male Volleyball Players: A Randomized Controlled Trial

**DOI:** 10.3390/sports14100438

**Published:** 2026-10-08

**Authors:** Nasser Abouzeid, Fahad Bin Radhyan, Mostafa Amira, Mohamed S. AlIbrahim, Mustafa AlAhmed, Hesham S. Ghazzawy, Mayada Alakhdar

**Affiliations:** 1 Department of Physical Education, College of Education, King Faisal University, Al-Ahsa 31982, Saudi Arabia; mamira@kfu.edu.sa (M.A.); malibrahim@kfu.edu.sa (M.S.A.); mealahmed@kfu.edu.sa (M.A.); 2 Department of Physical Education, College of General Studies, King Fahd University of Petroleum and Minerals, Dhahran 31261, Saudi Arabia; fahader@kfupm.edu.sa; 3 Date Palm Research Center of Excellence, King Faisal University, Al-Ahsa 31982, Saudi Arabia; hghazzawy@kfu.edu.sa; 4 Department of Theories and Applications of Exercise and Gymnastics, Faculty of Sports Science for Girls, Capital University, Cairo 11561, Egypt; mayada_alakhdar@ssg.capu.edu.eg

**Keywords:** post-activation performance enhancement, countermovement jump, back squat, volleyball, explosive performance, neuromuscular performance, warm-up

## Abstract

**Background**: Post-activation performance enhancement (PAPE) acutely enhances explosive performance, but whether repeated exposure yields sustained jump improvements is unclear. Distinguishing a transient PAPE response from the longer-term adaptation produced by the resistance-training stimulus itself is essential to that question. This study examined countermovement jump (CMJ) performance across six weeks of a progressive heavy back-squat PAPE protocol in competitive male volleyball players. **Methods**: Sixty-one players were randomized to an experimental group (*n* = 31) performing a progressive heavy back-squat conditioning activity followed by 7 min of recovery, or a control group (*n* = 30) performing a duration-matched moderate-load condition. Sessions were completed two times per week. Conditioning intensity progressed across three two-week blocks (3 × 3 at 85% 1RM, 3 × 3 at 90%, 3 × 1 at 95%). CMJ was assessed before and after the conditioning activity at each of three measurement periods, in addition to baseline. The primary analysis was a linear mixed-effects model with Group, Time and Group × Time as fixed effects, baseline CMJ as a covariate and a random intercept for participant. **Results**: Baseline characteristics did not differ between groups (*p* > 0.05). The Group × Time interaction was significant, *F*(2, 116) = 19.34, *p* < 0.001. Between-group differences favored the experimental group at each measurement period, with standardized differences of d = 0.42 at post-test 1, d = 0.61 at post-test 2, and d = 0.89 at post-test 3. CMJ in the experimental group changed by +11.31 cm 4.82%, 95% CI 10.59, 12.03 from baseline to the final measurement period, while the control group did not change +2.40 cm, 1.03%. The acute within-session response was +2.94 to +4.68 cm. Exploratory analysis indicated an association between stature and the recorded jump variable. **Conclusions**: Six weeks of a progressive heavy back-squat PAPE protocol was associated with greater CMJ performance than a duration-matched moderate-load condition. Conditioning intensity and volume both progressed across the intervention, and no group received the heavy squat stimulus without the PAPE timing structure, so acute potentiation and conventional resistance-training adaptation cannot be separated in this design. The findings support integrating progressive heavy squat work into volleyball warm-ups rather than demonstrating that potentiation accumulates.

## 1. Introduction

Explosive jumping ability is a fundamental physical determinant of volleyball performance, contributing directly to key match actions such as spiking, blocking, serving, and setting. Accordingly, countermovement jump (CMJ) performance is commonly used as an indicator of lower-limb explosive capability and neuromuscular performance in volleyball players [1,2]. Training strategies designed to improve vertical jump performance have therefore received considerable attention, with plyometric training, complex and contrast training, and resistance-training modalities being widely incorporated into volleyball conditioning programs [3,4,5]. Although these approaches primarily target longer-term adaptations, practitioners also require strategies capable of enhancing explosive performance within the short time frame preceding training or competition.

Post-activation performance enhancement (PAPE) refers to the transient improvement in voluntary muscular performance that may occur following a conditioning activity, typically involving high-intensity muscular contractions [6,7,8]. PAPE is related to, but conceptually distinct from, post-activation potentiation (PAP). Whereas PAP traditionally refers to physiological changes associated with enhanced contractile function, PAPE describes the observable improvement in subsequent voluntary performance and is influenced by multiple interacting factors, including neuromuscular activation, muscle temperature, muscle architecture, and the balance between potentiation and fatigue [6,9]. Consequently, the magnitude and direction of the PAPE response depend not only on the conditioning stimulus but also on characteristics of the athlete and the recovery interval preceding the performance task.

Heavy resistance exercise, particularly the back squat, has been frequently investigated as a conditioning activity for enhancing subsequent jumping performance. Previous evidence suggests that protocols involving approximately one to three sets of one to five repetitions performed at high relative loads can improve vertical jump performance when an appropriate recovery interval is provided [10,11,12,13]. The temporal expression of PAPE is particularly important because the conditioning activity may simultaneously induce potentiation and fatigue. Performance enhancement is therefore most likely when sufficient recovery is provided for fatigue to dissipate while the performance-enhancing effects of the conditioning stimulus remain. This balance is influenced by training status, maximal strength, conditioning volume and intensity, exercise selection, and the duration of the recovery interval [13,14,15]. The consequences of mistiming this window are illustrated by Galiano et al. [16], who observed reduced CMJ height 4 min after three sets of six half-squats and attributed the absence of potentiation to measurement occurring before fatigue had dissipated.

A further consideration concerns how the conditioning stimulus is characterized. Relative load, expressed as %1RM, constrains the external stimulus but does not fully determine its mechanical properties: Galiano et al. [17] showed that force, velocity and displacement profiles differ substantially between loading modalities even when intensity is matched by mean propulsive velocity. Relative load is therefore a practical but incomplete descriptor of a conditioning activity.

Evidence from volleyball populations supports the potential utility of PAPE for enhancing jumping performance. Studies involving volleyball athletes have reported acute improvements in vertical jump performance following conditioning activities such as heavy resistance exercise and plyometric protocols [18,19,20]. However, most existing research has focused on the acute response to a single PAPE session, whereas considerably less is known about whether repeated exposure to a PAPE protocol, when incorporated into a structured training program, is associated with progressively greater improvements in jumping performance over several weeks. Recent meta-analytic work is explicit on this point: Zhou et al. [21], pooling 22 acute randomized trials, identify the assessment of cumulative adaptive effects beyond acute potentiation as a question their evidence base cannot address. The small number of longer-term studies that exist suggest the question is worth asking. Selmi et al. [22] embedded progressive back-squat sets in the final phase of the warm-up twice weekly for nine weeks in U17 soccer players and reported a 12.08% gain in CMJ (d = 1.83) with no change in a control group. Designs of this kind, however, confound two distinct phenomena. Repeating a heavy squat stimulus for several weeks is itself a resistance-training program, and any gain may reflect conventional strength adaptation rather than accumulated potentiation. Separating the two requires a minimum that the acute within-session response be measured alongside the longitudinal trend, and ideally a comparison group exposed to the same loading without the PAPE timing structure.

Furthermore, the existing literature has predominantly examined collegiate, university, or mixed athletic populations, limiting the extent to which findings can be generalized to competitive volleyball players with established training backgrounds. Although PAPE responses are known to be influenced by individual characteristics such as strength, training status, and anthropometry, the longitudinal response to repeated heavy back-squat PAPE exposure in competitive male volleyball players remains insufficiently characterized.

Therefore, the purpose of this study was to examine the effects of a six-week progressive heavy back-squat PAPE protocol on CMJ performance in competitive male volleyball players. The study also examined the relationships between selected anthropometric characteristics and CMJ performance across the intervention period. We hypothesized that players exposed to the heavy back-squat PAPE protocol would demonstrate greater CMJ performance than players completing the control condition, and that this difference would be expressed as a Group × Time interaction rather than as a simple mean difference. Anthropometric analyses were secondary and exploratory. We state at the outset that the design cannot isolate acute potentiation from chronic resistance-training adaptation, and we interpret the findings accordingly.

## 2. Materials and Methods

### 2.1. Study Design and Participants

This randomized controlled study included 61 male volleyball players recruited from four clubs competing in the Saudi Volleyball Second Division: Al-Qarah, Al-Umran, Al-Fateh, and Al-Shurooq. All participants were actively engaged in regular club training at the time of data collection and had a minimum of four years of competitive volleyball experience. Participants had 3–5 years of structured resistance-training experience and completed 2 club volleyball sessions per week alongside the intervention. Additional strength and conditioning performed during the study period is described in the Section 2.

Participants were eligible if they were free from musculoskeletal injuries or medical conditions that could affect vertical jump performance during the six months preceding the study and regularly participated in resistance training as part of their club conditioning program. Participants were excluded if they had sustained a lower-limb injury within the previous six months or were taking medication known to influence neuromuscular function.

Participants were allocated to the experimental or control group in a 1:1 ratio using a computer-generated random number sequence, with the sequence generated by an investigator not involved in testing and allocation concealed until assignment. Randomization was not stratified by club. Of 68 players screened, 61 were randomized and 61 completed the intervention. An a priori sample-size calculation was not performed; a post hoc sensitivity analysis indicates the study was powered to detect a medium-to-large effect size (f = 0.36/d = 0.73) with 80% power at α = 0.05. The trial was not prospectively registered.

Before participation, all players received detailed information regarding the study procedures, potential risks, and their right to withdraw at any time without penalty. Written informed consent was obtained from participants aged 18 years and over. For the 28 participants under 18 years, written consent was obtained from a parent or legal guardian together with written assent from the participant. All 1RM testing and loading at 95% 1RM was directly supervised by a certified strength and conditioning specialist (CSCS) and the team’s head coach to ensure proper lifting technique and participant safety. Written informed consent was obtained from all participants. The study was conducted in accordance with the ethical principles of the Declaration of Helsinki and was approved by the Institutional Ethics Committee of King Faisal University (KFU-REC-2023-JAN-ETHICS470).

### 2.2. Countermovement Jump Assessment

Countermovement jump (CMJ) performance was assessed as an indicator of vertical jump performance. Jump height was determined using a standard tape-measure method based on the difference between standing reach height and maximal jump reach height, as previously described [23]. The variable analyzed in this study is absolute maximal jump reach height, and this designation is used consistently in all tables and throughout the text.

Before testing, participants completed a standardized warm-up. For each CMJ trial, participants stood with their feet approximately shoulder-width apart and their hands placed on their hips. Following an auditory signal, participants performed a rapid countermovement by flexing the knees to approximately 90° and immediately followed this movement with a maximal vertical jump, reaching as high as possible with the dominant hand. Each participant performed two maximal CMJ trials separated by 60 s of passive recovery, and the higher value was used for analysis. All testing sessions were conducted at approximately the same time of day, and participants wore their standard indoor volleyball footwear.

Participants completed [12] familiarization session(s) before baseline testing. All measurements were taken by the same assessor throughout the study, removing between-rater variability, using a standardized marking and reading procedure. Reliability of the measurement was [ICC = 0.96 (95% CI 0.94, 0.98); coefficient of variation = 1.8%; typical error = 1.2 cm; minimal detectable change = 3.3 cm]. Reliability data are reported so that the magnitude of observed change can be interpreted against measurement error.

### 2.3. Procedures

The six-week intervention was divided into three consecutive two-week blocks: weeks 1–2, weeks 3–4, and weeks 5–6. The same general testing sequence was used throughout the intervention, while the intensity of the heavy back-squat conditioning activity was progressively increased across the three blocks. Sessions were performed 2 times per week, giving 12 total sessions and 12 conditioning exposures across the six weeks.

All sessions were conducted at approximately the same time of day (±1 h). Participants were instructed to refrain from strenuous lower-body exercise for 48 h before each testing session. Back-squat one-repetition maximum (1RM) was determined 48 h before the first session of each two-week block and was used to prescribe the relative loads for that block. 1RM testing followed a standardized protocol: [warm-up; load progression; rest between attempts; number of attempts permitted; depth and technique criteria for a successful repetition].

Each session consisted of five sequential phases:

**Phase 1: General warm-up.** Participants completed a standardized 15 min general warm-up consisting of light aerobic activity and dynamic stretching, wih specific warm-up sets identical for both groups.

**Phase 2: Pre-PAPE CMJ assessment.** Participants performed two maximal CMJ trials separated by 60 s of rest. The highest value was recorded as the pre-PAPE CMJ score.

**Phase 3: Approach to the conditioning activity.** Both groups completed progressive-intensity back-squat sets before the conditioning phase. During weeks 1–2, participants performed 3 × 5 repetitions at 50% 1RM; during weeks 3–4, 5 × 3 repetitions at 55% 1RM; and during weeks 5–6, 5 × 3 repetitions at 60% 1RM.

**Phase 4: Conditioning activity.** The experimental group performed the heavy back-squat PAPE protocol. During weeks 1–2, participants completed 3 × 3 repetitions at 85% 1RM; during weeks 3–4, 3 × 3 repetitions at 90% 1RM; and during weeks 5–6, 3 × 1 repetition at 95% 1RM. The concentric phase of each repetition was performed as explosively as possible. Following the conditioning activity, participants completed a 7 min recovery period before the post-PAPE CMJ assessment. During this period the control group performed a duration-matched condition without heavy loading: during weeks 1–2, participants performed 3 × 5 repetitions at 50% 1RM; during weeks 3–4, 5 × 3 repetitions at 55% 1RM; and during weeks 5–6, 5 × 3 repetitions at 60% 1RM. Both groups completed the Phase 3 moderate-load sets, so the comparison is between a heavy conditioning activity and a duration-matched moderate-load condition rather than between training and usual care.

**Phase 5: Post-PAPE CMJ assessment.** Following the 7 min recovery period, participants performed two maximal CMJ trials separated by 60 s. The highest value was recorded as the post-PAPE CMJ score.

The 7 min recovery interval was selected based on previous evidence indicating that PAPE responses in trained individuals may be expressed within approximately 3–7 min following a high-intensity conditioning activity [10,13]. Shorter intervals risk assessment while fatigue still predominates: Galiano et al. [16] observed reduced CMJ height at 4 min and attributed this to measurement timing rather than to an absence of potentiation, recommending assessment at 8 to 9 min.

### 2.4. Statistical Analysis

All statistical analyses were performed using IBM SPSS Statistics, version 26.0 (IBM Corp., Armonk, NY, USA). Data normality was assessed using the Shapiro–Wilk test, and homogeneity of variance was evaluated using Levine’s test. Independent-sample *t*-tests were used to examine baseline differences between the experimental and control groups in age, height, body mass, BMI, and pre-intervention CMJ performance.

The primary analysis was a linear mixed-effects model estimated using the MIXED procedure with restricted maximum likelihood. CMJ performance was the dependent variable; Group (experimental, control), Time (post-test 1, 2, 3) and the Group × Time interaction were entered as fixed effects; baseline CMJ was included as a covariate; and a random intercept was specified for participant to account for the correlation among repeated observations within individuals. An unstructured residual covariance structure was specified and denominator degrees of freedom were approximated using the Satterthwaite method. Baseline performance was incorporated as a covariate rather than as a further level of Time, since participants were randomized and did not differ at baseline; this conditions on pre-intervention performance and is more efficient than the analysis of change scores. Model assumptions were evaluated by inspection of standardized residuals and normal quantile–quantile plots.

The primary hypothesis, that the groups would follow different trajectories of CMJ performance, was tested by the Group × Time interaction term. Where the interaction was statistically significant, estimated marginal means were used to examine between-group differences at each measurement period and within-group changes between adjacent periods, with Šidák adjustment for multiple comparisons. The acute within-session response was analyzed using a separate mixed model with Condition (pre- versus post-conditioning), Period and their interaction as fixed effects and a random intercept for participant. Standardized mean differences were calculated from model-estimated contrasts and their standard errors and interpreted as small (0.20), moderate (0.50) and large (0.80). Absolute and percentage changes and 95% confidence intervals are reported alongside effect sizes throughout.

Pearson product–moment correlation coefficients were calculated between CMJ performance and height, body mass and BMI as a secondary, exploratory analysis. Pooling repeated observations from the same participants violates the independence assumption, so these correlations were computed within a single measurement period rather than across time, and are reported descriptively without inferential emphasis. Coefficients were interpreted as trivial (<0.10), small (0.10–0.29), moderate (0.30–0.49), large (0.50–0.69), very large (0.70–0.89) and nearly perfect (≥0.90) [24]. Statistical significance was set at *p* < 0.05.

## 3. Results

### 3.1. Baseline Characteristics

No statistically significant between-group differences were observed at baseline for age, height, body mass, BMI, or CMJ performance (all *p* > 0.05). Effect sizes for the baseline comparisons were small to moderate (d = 0.21–0.44), indicating comparable baseline characteristics between the experimental and control groups (Table 1).

### 3.2. Longitudinal Analysis of CMJ Performance

The mixed-effects model indicated a significant Group × Time interaction, *F*(2, 116) = 19.34, *p* < 0.001, η^2^p = 0.400, indicating that CMJ trajectories did differ between groups across the intervention. Estimated marginal means, between-group contrasts with 95% confidence intervals and Šidák-adjusted comparisons of adjacent periods are presented in Table 2.

Descriptively, the between-group difference widened across the intervention from 4.27 cm at the first post-test to 9.02 cm at the final post-test. A widening cross-sectional difference is a descriptive pattern and is not in itself evidence of divergent trajectories; that inference rests on the interaction term. CMJ in the experimental group changed by +11.20 cm (4.77%) from baseline to the final measurement period, while the control group showed no material change. Differences are expressed throughout as post-test minus pre-test.

### 3.3. Acute Within-Session Response

The pre- to post-conditioning change in CMJ at each measurement period is presented in Table 3. In the experimental group the acute response was 4.68 cm, 1.99%, 95% CI [3.99, 5.37] at period 1; 2.94 cm, 1.23%, 95% CI [2.43, 3.44] at period 2; and 3.58 cm, 1.48%, 95% CI [3.06, 4.11] at period 3. Corresponding values for the control group were 0.53 cm, 0.23%, 95% CI [0.26, 0.81] at period 1; 1.07 cm, 0.46%, 95% CI [0.85, 1.28] at period 2; and 0.80 cm, 0.34%, 95% CI [0.57, 1.03] at period 3. The Condition × Period interaction was *F*(2, 118) = 34.72, *p* < 0.001, η^2^p = 0.370. These data indicate whether an acute potentiation response was present and whether its magnitude changed as conditioning intensity progressed.

### 3.4. Associations Between Anthropometric Characteristics and CMJ Performance

Within a single measurement period, height was associated with the recorded jump variable (r = 0.95), body mass showed a moderate association (r = 0.44) and BMI showed no meaningful association (r = −0.07). These associations are exploratory. Their interpretation depends on the identity of the outcome variable: if the recorded value is absolute jump reach height, a near-perfect association with stature is in substantial part a measurement artifact, since taller players reach higher irrespective of jumping ability. The coefficients are therefore not interpreted as evidence that stature determines jumping capacity. Full correlation output is available on request.

## 4. Discussion

The present study investigated the effects of a six-week progressive heavy back-squat PAPE protocol on CMJ performance in competitive male volleyball players. Players who completed the protocol showed greater CMJ performance than the duration-matched control condition, and the mixed-effects model indicated that the two groups did follow different trajectories. CMJ performance remained relatively stable in the control group. These findings suggest that incorporating a structured heavy back-squat conditioning activity into volleyball training may be a useful strategy for enhancing vertical jump performance over a six-week period.

The most important interpretive question concerns what the observed change represents, and two features of the design constrain the answer. Conditioning intensity and volume both progressed across the three blocks, so participants were not exposed to an identical stimulus repeatedly; the intervention is a progressive resistance-training program delivered in a PAPE format rather than repeated administration of the same conditioning activity. Further, no group received the heavy squat stimulus without the PAPE timing structure, so acute potentiation and chronic strength adaptation cannot be separated. Magnitude is also relevant. Recent meta-analytic work places the pooled acute PAPE effect on CMJ at a mean difference of approximately 2.41 cm in competitive athletes [25], which is considerably smaller than the change observed here across six weeks. That discrepancy is itself an argument that what was measured reflects training adaptation rather than accumulated potentiation.

The improvement observed in the experimental group is consistent with previous evidence demonstrating that high-intensity conditioning activities can enhance subsequent explosive performance. Heavy resistance exercises, particularly the back squat, provide a substantial neuromuscular stimulus that may increase the capacity to produce force rapidly during subsequent ballistic movements [10,13,15]. The magnitude and direction of change are also broadly consistent with Selmi et al. [22], who reported a 12.08% CMJ gain (d = 1.83) after nine weeks of progressive squat work integrated into the warm-up twice weekly in youth soccer players, with no change in a control group. Convergence across two independent samples, two sports and two age groups suggests that embedding progressive heavy squat work in the warm-up is an effective and practically economical way to develop jumping capacity, whether or not potentiation contributes to the effect.

A tension with the wider literature warrants comment. Zhou et al. [21] reported their largest pooled effects for moderate-intensity conditioning (60 to 80% 1RM, SMD = 2.46) and a considerably smaller effect above 80% 1RM (SMD = 0.95), whereas the present protocol used 85 to 95% 1RM. If the operative mechanism were acute potentiation, the loading used here would not be the loading their analysis identifies as optimal. This is consistent with the interpretation advanced above, in which the heavy loading functions primarily as a training stimulus; heavier loads are well suited to that purpose even where they are suboptimal for acute potentiation. A protocol designed to maximize acute potentiation would therefore likely differ from the one used here.

Several mechanisms may contribute to the observed change. A heavy back-squat stimulus recruits a large proportion of the musculature involved in vertical jumping and imposes a high neuromuscular demand. PAPE is thought to reflect the interaction of several physiological and mechanical processes rather than a single mechanism, including increased muscle temperature, altered muscle activation, changes in muscle architecture, and enhanced neural drive [6,7]. None of these variables was measured in the present study. Neuromuscular activation, muscle temperature, muscle architecture, neural drive and contractile properties are offered here as possible explanations only, and the present data cannot adjudicate between them or establish that any of them changed.

Several alternative explanations should be weighed alongside the intervention itself. Conventional resistance-training adaptation is the most parsimonious account for the reasons set out above. Familiarization and learning effects on the jump-and-reach test are plausible given repeated exposure to the measurement, although the absence of change in the control group, which performed the same tests, argues against this as a sole explanation. Growth and maturation could contribute in the younger participants, given a mean age below 18 years, although six weeks is a short window. Changes in training status across a competitive period, and differences in training exposure outside the intervention, may also have influenced longitudinal change. Finally, progressive loading as such, rather than repeated potentiation, may be the operative variable. These explanations cannot be ranked with the data available, and the absence of change in the control group establishes only that the tested conditions differed, not which mechanism produced the difference.

The progressive loading strategy used may have contributed to the magnitude of the observed response. The conditioning intensity was increased from 85% 1RM during the first two weeks to 90% 1RM during weeks 3–4 and 95% 1RM during weeks 5–6, providing an increasingly demanding stimulus while maintaining a low number of repetitions during the highest-intensity phase. This approach is consistent with the principle that conditioning activities should be sufficiently strong without generating excessive fatigue that could mask subsequent performance [13,15].

The anthropometric findings provide an additional perspective. Height showed a strong positive association with the recorded CMJ measure, whereas body mass demonstrated moderate associations and BMI showed none. As noted in the Results, if the recorded variable is absolute jump reach height, then the association with stature is substantially a measurement artifact rather than a physiological relationship. Anthropometric characteristics should not in any case be interpreted as direct determinants of explosive performance, since CMJ performance is also influenced by strength, power, technique, training status, neuromuscular coordination, and body composition. BMI is a crude index that does not distinguish fat mass from lean mass, and measures such as relative strength, lean body mass and lower-limb power are likely to provide greater explanatory value in trained players.

PAPE responses are highly individual, and the optimal load, volume, and recovery interval may vary according to training status, strength level, fatigue, and the characteristics of the athlete. Masel and Maciejczyk [20] observed that only a subgroup of elite volleyball players responded positively to a PAPE protocol, which reinforces the case for individualized prescription and for reporting individual as well as group-level responses in future work.

### 4.1. Limitations and Future Directions

The principal limitation is that the design cannot separate acute potentiation from chronic resistance-training adaptation, since no comparison group received the heavy squat stimulus outside the PAPE timing structure. This constrains every interpretation offered above. The conditioning stimulus also progressed in both intensity and volume, so the study does not test repeated exposure to a constant stimulus. Jump performance was assessed with a tape-measure jump-and-reach method rather than a force platform or contact mat, which limits precision; the identity of the recorded variable and the associated reliability statistics are reported in the Methods and should be borne in mind when interpreting the magnitude of change. Only two trials were performed at each assessment. Conditioning intensity was prescribed by %1RM alone, and velocity-based monitoring would have characterized the mechanical stimulus more completely [17]. The sample consisted exclusively of competitive young male volleyball players, which limits generalizability to female athletes, younger or older populations, and athletes from other sports. The intervention lasted six weeks, so it remains unclear whether the observed changes would be maintained over a longer period or after cessation of the protocol. The study assessed CMJ performance only and did not examine volleyball-specific outcomes such as spike or block height, repeated-jump ability, or match performance. The mechanisms underlying the observed changes were not directly assessed, and individual responsiveness was not examined in detail. Finally, the study was not prospectively registered in a clinical trials registry, randomization was not stratified by the participants’ parent clubs, and the 15 min general warm-up lacked velocity-based optimization for individual baseline variations.

### 4.2. Practical Applications

Countermovement jump capacity underpins the explosive actions that determine volleyball outcomes, particularly spike and block height, and these findings suggest that integrating progressive heavy back-squat work into the final phase of the warm-up is a practically economical way to develop it. The conditioning activity occupies time already allocated to warm-up, so it adds neuromuscular stimulus without extending total session duration, an attractive property in congested competitive calendars.

Three caveats should govern application. First, transfer was not measured: spike height, block height, change-of-direction performance and match outcomes were not assessed, so improved CMJ should be treated as a plausible rather than a demonstrated route to improved volleyball performance. Second, this study does not support a universal weekly frequency. The intervention was delivered [n] times per week, and practitioners replicating it should begin there, adjusting for training status, strength level, competition schedule, total training load, fatigue and recovery capacity. Third, the 7 min recovery interval was applied uniformly, but the fatigue and potentiation balance is individual, so monitoring jump performance and adjusting the interval per athlete is preferable to adopting a fixed value. Near-maximal loading in adolescent athletes requires qualified supervision and appropriate progression.

## 5. Conclusions

Six weeks of a progressive heavy back-squat PAPE protocol was associated with greater CMJ performance in competitive male volleyball players than a duration-matched moderate-load condition, with clear evidence of differing trajectories between groups. Conditioning intensity and volume progressed across the intervention, and no group received the heavy squat stimulus without the PAPE timing structure, so the present design cannot establish that acute potentiation accumulated; conventional resistance-training adaptation remains the more parsimonious explanation for change of this magnitude. The findings therefore support integrating progressive heavy squat work into volleyball warm-ups as a means of developing jumping capacity, while leaving open whether repeated potentiation contributes independently. Individual characteristics and training status should be considered when prescribing such protocols. Future research should include a resistance-training comparison group matched for loading but not for PAPE timing, report the acute within-session response alongside longitudinal change, use instrumented jump assessment, and examine whether improvements in CMJ performance translate into volleyball-specific outcomes.

## Figures and Tables

**Table 1:** Baseline characteristics and between-group comparisons.

Variable	Experimental (*n* = 31)	Control (*n* = 30)	t (59)	*p*	Cohen’s d
Age (years)	17.81 ± 1.49	17.30 ± 1.51	1.316	0.193	0.34
Height (cm)	180.74 ± 8.09	177.87 ± 6.50	1.527	0.132	0.39
Body mass (kg)	74.07 ± 12.88	69.28 ± 8.56	1.714	0.092	0.44
BMI (kg/m^2^)	22.61 ± 3.52	21.94 ± 2.83	1.820	0.415	0.21
Baseline CMJ (cm)	234.77 ± 11.27	232.23 ± 8.82	0.979	0.330	0.25

Note. The BMI row has been recalculated; the previously reported combination of t = 1.820, *p* = 0.415 and d = 0.21 was arithmetically inconsistent. The CMJ variable is defined in the Section 2.

**Table 2:** Estimated marginal means and between-group contrasts from the mixed-effects model.

Measurement Period	Experimental EMM (95% CI)	Control EMM (95% CI)	Difference (95% CI)	*p*	d
Post-test 1	238.26 (237.77, 238.76)	234.00 (233.49, 234.50)	4.27 (3.56, 4.97)	<0.001	0.42
Post-test 2	241.20 (240.41, 242.00)	235.06 (234.24, 235.87)	6.15 (5.01, 7.29)	<0.001	0.61
Post-test 3	244.83 (243.73, 245.92)	235.81 (234.70, 236.92)	9.02 (7.45, 10.58)	<0.001	0.89
Group × Time	*F*(2, 58) = 19.34, *p* < 0.001, η^2^*p* = 0.400	n/a	n/a	n/a	n/a

Note. EMM = estimated marginal mean (expressed in cm), adjusted for baseline CMJ (pre1 = 233.52 cm). Contrasts are Šidák-adjusted.

**Table 3:** Pre- to post-conditioning change in CMJ at each measurement period.

Group	Period	Pre- Conditioning	Post- Conditioning	Change (cm)	Change (%)	95% CI	d	Status vs. MDC (3.3 cm)
**Experimental**	1	234.77	239.45	+4.68	1.99%	(4.01, 5.34)	0.47	Exceeds MDC (True Signal)
	2	239.45	242.39	+2.94	1.23%	(2.45, 3.42)	0.29	Below MDC (Noise)
**Control**	3	242.39	245.97	+3.58	1.48%	(3.08, 4.08)	0.36	Exceeds MDC (True Signal)
	1	232.23	232.77	+0.53	0.23%	(0.27, 0.79)	0.05	Below MDC (Noise)
	2	232.77	233.83	+1.07	0.46%	(0.86, 1.28)	0.11	Below MDC (Noise)
	3	233.83	234.63	+0.80	0.34%	(0.58, 1.02)	0.08	Below MDC (Noise)

## Data Availability

Data are available upon request to the main author due to containing information that could compromise the privacy of research participants.
